# Extended Durability of a Cloth-Covered Star-Edwards Caged Ball Prosthesis in Aortic Position

**DOI:** 10.1155/2009/165858

**Published:** 2010-02-03

**Authors:** Yusuf Ata, Tamer Turk, Cüneyt Eris, Mihriban Yalcin, Filiz Ata, A. Ozyazicioğlu

**Affiliations:** ^1^Department of Cardiovascular Surgery, Bursa Yuksek Ihtisas Education and Research Hospital, 16330 Bursa, Turkey; ^2^Department of Anesthesiology, Bursa Yuksek Ihtisas Education and Research Hospital, 16330 Bursa, Turkey

## Abstract

The Starr-Edwards caged ball valve is one of the oldest cardiac valve prosthesis and was widely used all around the world in the past decades. Despite the long-term results that have been reported there are only a few cases reported that exceed 30 years of durability. Here in, we report a 53-year-old patient with a well-functioning 35-year-old aortic Starr-Edwards caged ball prosthesis.

## 1. Introduction

The Starr-Edwards caged ball heart valve marked a new era in the treatment of valvular heart disease. Complications including thrombosis, thromboebolic events, paravalvular leakage, and pannus formation that occurred in variable frequencies were reported regarding to this artificial valve. Several modifications of Starr-Edwards valves were implanted over 200,000 cases all round the world, and long-term results with these valves have been reported showing satisfactory results with reliable durability and safety [1–3]. For elimination of the larger metal surface and variance in the original silastic ball and aiming to reduce thromboembolism, cloth-covered model of the Starr-Edwards stellite-ball valve was introduced. We report a 53-year-old patient with well functioning cloth-covered Star-Edwards caged ball prosthesis implanted 35-years-ago.

## 2. Case Report

**Figure 1:**
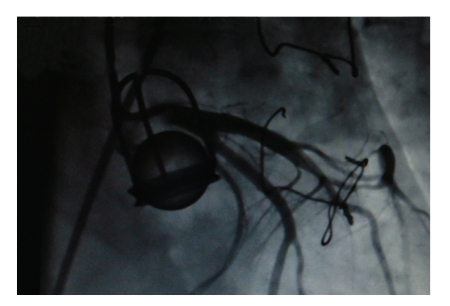
Angiography showing the functioning Star-Edwards Caged ball prosthesis at diastol.

**Figure 2:**
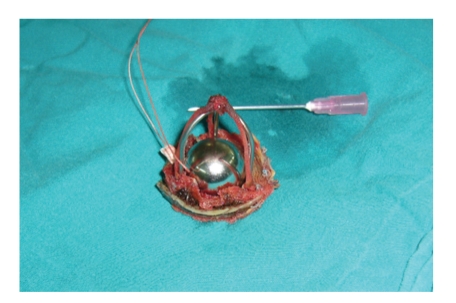
Significant cloth wear and dislodgement and bare cage metal of the excised caged ball prosthesis.

A-53-year old man admitted to our institution with exertional dyspnea and angina. He had had aortic valve replacement with Starr-Edwards 2320 model caged ball mechanical valve prosthesis in 1974 and suffered a thromboembolic serebrovascular accident two years ago. His physical examination revealed left hemiparesis with blood pressure 120/80 mmHg, heart rate 92 beats/min and regular. Cardiac auscultation revealed a 4/6 systolic murmur and rales were heard at the base of the lungs. Transesophageal echocardiography showed a functioning Starr-Edwards caged ball mechanical prosthesis with 30 mmHg gradient and rheumatic mitral valve disease with peak transmitral gradient of 20 mmHg and valve area 1.8 cm^2^ and 3-4 degree mitral insufficiency with a left ventricular ejection fraction of 30% with peak pulmonary artery pressure of 68 mmHg, besides there was a pannus formation in the surrounding valvular tissue. Coronary arteriography showed a 90% stenosis at proximal portion of LAD and well functioning caged ball prosthesis (Figure 1). The patient underwent a reoperation of double valve replacement and one vessel coronary artery bypass grafting through a median sternotomy by the help of cardiopulmonary bypass at mild hypothermia. Macroscopic inspection of the excised Starr-Edwards caged ball significant cloth wear and dislodgement were observed inside the struts, showing bare cage metal (Figure 2).

At reoperation the mitral valve was replaced with a bileaflet prosthesis, St. Jude Medical 29 mm, and an aortotomy was applied because of the previous serebrovascular accident and to examine the pannus formation in the surrounding tissue. After aortotomy we saw that the cloth surrounding the outside was detached from struts and loosened and the Starr-Edwards caged-ball prosthesis was replaced with St. Jude Medical 21 mm bileaflet valve prosthesis to achieve a better hemodynamic performance and a lower INR ratio in the follow-up period. The postoperative course was uneventful. Echocardiogram before discharge confirmed that the prosthetic valves were functioning normally.

## 3. Discussion

The first Starr-Edwards caged-ball valve was used in mitral position in 1960 and was introduced by Albert Starr in 1961 [1]. Despite complications such as high-pressure gradient, paravalvular leakage, pannus formation, thrombosis and thoromboembolic events that occur in variable frequencies, this artificial valve have been used worldwide in the past decades. With reported long term durability and good hemodynamic performance approximately 200,000 Starr-Edwards caged ball valves with several modifications have been implanted [2–6]. Nevertheless potential complications of this artificial valve remained a permanent problem. The design of the valve leads to absence of central flow and allows only a lateral flow which results in higher transvalvular gradients. Therefore, the combination of nonphysiological surfaces of the valve and stasis due to higher gradients creates a predisposition for fibrous pannus or thrombus formation. Thrombosis and pannus formation mostly occur at the apex or at the base of the cage either leading to stenosis or stuck valve.

Some authors reported evidence of ball variance for Starr-Edwards caged ball prosthesis [7, 8]. In our case the patient had still normally functioning Starr-Edwards caged ball valve but at the excision time the cloth surrounding the outside was detached from struts and loosened. Structural valve deterioration and thromboembolism rates were reported to be higher in aortic group by Godje and colleagues who performed a study in patients who had aortic or mitral valve replacement with Starr-Edwards caged ball prosthesis [3]. 

Here we report a patient in whom Starr-Edwards caged ball prosthesis functioned well for 35 years and was still functioning well although structurally cloth-cover of the prosthesis was worn. We replaced this functioning valve with a bileaflet mechanical valve prophylactically to achieve a better hemodynamic performance and for a lower INR ratio in the follow-up period.

There are a few patients reported to have lived with an aortic Starr-Edwards caged ball prosthesis over 30 years or longer especially without mechanical or hemodynamical deterioration [3–10]. Therefore survival for 35 years with still normally functioning aortic Starr-Edwards caged ball prosthesis is noteworthy. 

## References

[1] Starr A, Edwards ML (1961). Mitral replacement: clinical experience with a ball-valve prosthesis. *Annals of Surgery*.

[2] Cobanoglu A, Starr A (1987). Starr-Edwards silastic ball prosthesis: state of the art 25 years later. *Cardiac Surgery: State of the Art Reviews*.

[3] Gödje OL, Fischlein T, Adelhard K, Nollert G, Klinner W, Reichart B (1997). Thirty-year results of Starr-Edwards prostheses in the aortic and mitral position. *Annals of Thoracic Surgery*.

[4] Lazaros GA, Dounis GB, Panou FC, Georgoulas VP, Zacharoulis AA (1998). Thirty one-year durability of a Starr-Edwards aortic prosthesis. *Journal of Cardiovascular Surgery*.

[5] Ozkokeli M, Ates M, Ekinci A, Akcar M (2005). Thirty-seven-year durability of a Starr-Edwards aortic prosthesis: case report and brief review of the literature. *Texas Heart Institute Journal*.

[6] Ikizler M, Birdane A, Sevin B (2007). An old friend is still at work: 34-year-old well functioning Starr-Edwards aortic prosthesis without anticoagulation. *International Journal of Cardiology*.

[7] Grunkemeier GL, Starr A (1986). Late ball variance with the model 1000 Starr-Edwards aortic valve prosthesis: risk analysis and strategy of operative management. *Journal of Thoracic and Cardiovascular Surgery*.

[8] Peterman MA, Donsky MS, Matter GJ, Roberts WC (2006). A Starr-Edwards model 6120 mechanical prosthesis in the mitral valve position for 38 years. *American Journal of Cardiology*.

[9] Bessell JR, Gower G, Craddock DR, Stubberfield J, Maddern GJ (1996). Thirty years experience with heart valve surgery: isolated aortic valve replacement. *Australian and New Zealand Journal of Surgery*.

[10] Tarzia V, Bottio T, Testolin L, Gerosa G (2007). Extended (31 years) durability of a Starr-Edwards prosthesis in mitral position. *Interactive Cardiovascular and Thoracic Surgery*.

